# Bridging the Knowledge–Practice Gap in Cervical Spine Injury First Aid: A Cross-Sectional Study in Southern Saudi Arabia

**DOI:** 10.3390/healthcare14091241

**Published:** 2026-05-04

**Authors:** Yahya H. Khormi, Mohammad A. Jareebi, Ali Y. Madkhali, Nasser A. N. Abu Alzawayid, Amjad H. Muthaffar, Taif A. Masri, Eyad M. Albarrati, Mohammed H. Hakami, Suha Ali Ageeli, Mohammed S. Alshahrani, Ruba M. Alzahrani, Faisal H. Tawashi, Ibrahim A. Hakami, Ghazi I. Al Jowf, Farjah H. Algahtani

**Affiliations:** 1 Department of Surgery, College of Medicine, Jazan University, Jazan 45142, Saudi Arabia; khormins@gmail.com; 2 Family and Community Medicine Department, College of Medicine, Jazan University, Jazan 45142, Saudi Arabia; 3 Faculty of Medicine, Jazan University, Jazan 45142, Saudi Arabia; ali27yahyamad@gmail.com (A.Y.M.); n.azawayid@gmail.com (N.A.N.A.A.); amjadhasan746@gmail.com (A.H.M.); taif01masri@gmail.com (T.A.M.); eyad.albarrati@gmail.com (E.M.A.); mo7a.hak@gmail.com (M.H.H.); suha.ageeli8@gmail.com (S.A.A.); f.ht220@gmail.com (F.H.T.); 4 Faculty of Medicine, King Khalid University, Abha 61421, Saudi Arabia; m00093065@gmail.com; 5 Faculty of Medicine, Al-Baha University, Al-Baha 65522, Saudi Arabia; roubaalzahrani@gmail.com; 6 Orthopedic Surgery, Faculty of Medicine, Shaqra University, Dawadmi 15248, Saudi Arabia; ihakami@su.edu.sa; 7 Department of Public Health, College of Applied Medical Sciences, University Medical Clinics Complex, King Faisal University, Al Hofuf 37912, Saudi Arabia; galjowf@kfu.edu.sa; 8 Oncology Center, Chair of Epidemiology and Public Health Research, Faculty of Medicine, King Saud University/King Saud Medical City, Riyadh 12373, Saudi Arabia; falgahtani@ksu.edu.sa

**Keywords:** cervical spine injury, first aid, trauma awareness, emergency response, road traffic accidents, spinal immobilization, injury prevention, public health

## Abstract

Background/Objectives: Cervical spine injuries (CSIs) are life-threatening conditions commonly resulting from road traffic accidents and falls; improper first aid management can significantly worsen neurological outcomes. Public awareness and correct first aid response are critical for preventing secondary injury; despite this, available data from the southern provinces of Saudi Arabia remain insufficient. This study aimed to assess public awareness and first aid preparedness for CSIs across four southern regions of Saudi Arabia. Methods: A cross-sectional design was employed across multiple regions, encompassing 1179 adults from Jazan, Aseer, Al-Baha, and Najran between 2025 and 2026. A validated online questionnaire was employed for data collection to assess CSI awareness, recognition of injury signs, and appropriate first aid responses. Awareness scores of ≥60% were classified as good. Multiple linear regression analysis was performed to identify independent predictors of awareness. Results: The mean awareness score was 16.0 ± 4.8 out of a possible total of 20 points, corresponding to 80% of the total score, with 87% of participants demonstrating good awareness. The majority of respondents recognized the importance of spinal immobilization (89%), maintaining head–neck alignment (95%), and contacting emergency services before intervention (93%). Correct responses to emergency scenarios were recorded in 83% of participants. Notably, only 39% had attended formal medical or trauma training, and merely 3% reported real-life first aid experience. Training attendance (β = 1.39, *p* < 0.001) and marital status (married; β = 1.37, *p* = 0.004) were identified as independent predictors of higher awareness scores. Conclusions: Although general public awareness of CSI first aid is high, formal training participation remains critically low, revealing a substantial gap between knowledge and practice. Integrating structured first aid training into community, workplace, and primary healthcare settings is essential to improve trauma outcomes and reduce preventable disability.

## 1. Introduction

Cervical spine injuries (CSIs) represent a critical public health concern globally, with profound implications for mortality, permanent disability, and quality of life. These injuries predominantly result from high-energy trauma, including road traffic accidents (RTAs), falls from heights, sports-related incidents, and industrial injuries [1,2]. The cervical spine, comprising seven vertebrae that support and protect the spinal cord while enabling head movement, is particularly vulnerable to traumatic injury due to its anatomical characteristics: high mobility, relatively smaller vertebral bodies, and weaker muscular support compared to thoracic and lumbar regions [3,4]. Injury mechanisms include flexion, extension, rotation, axial loading, and direct compression, each potentially resulting in devastating neurological sequelae [5].

The global epidemiology of traumatic spinal cord injuries is sobering. Recent global estimates report a traumatic spinal cord injury incidence of 26.48 per million people [6], and approximately 14.5 million people worldwide were living with spinal cord injury as of 2021, with RTAs and falls accounting for the majority of cases [7]. Saudi Arabia faces a particularly severe burden of road traffic trauma. RTAs rank as the second leading cause of death after infectious diseases, with motor vehicle collisions resulting in approximately one fatality and four injuries every hour [8,9,10,11]. This epidemic of road trauma has prompted national attention to injury prevention strategies, yet pre-hospital care capabilities—particularly public first aid competency—remain inadequately developed [12,13].

Appropriate pre-hospital management of suspected CSI is paramount to preventing secondary neurological damage. Improper handling, including excessive movement, inadequate immobilization, or delayed emergency medical services (EMS) activation, can convert incomplete spinal cord injuries to complete lesions, resulting in permanent paralysis or death [14,15]. Evidence-based first aid for CSI emphasizes three critical principles: (1) immediate recognition of injury mechanisms and clinical signs, (2) spinal motion restriction through manual stabilization and avoidance of unnecessary movement, and (3) early activation of professional EMS [16,17]. Despite the established importance of these interventions, international studies consistently demonstrate significant gaps in public knowledge and preparedness [14,18].

Previous research in Saudi Arabia has yielded inconsistent findings regarding CSI awareness. Findings from a study in Dammam demonstrated that 90.4% of participants possessed adequate knowledge of CSIs, with healthcare sector workers demonstrating superior awareness [19]. Conversely, a Makkah-based investigation found that only 53.8% of respondents had good awareness of appropriate CSI care [20]. Al-Otaibi et al. reported that fewer than one-third of participants nationwide were aware of CSIs or appropriate first aid approaches [1]. These discrepancies may reflect regional variations in educational exposure, healthcare access, or cultural factors influencing health literacy. Methodological differences across studies, including variations in sampling strategies, questionnaire instruments, scoring thresholds, and study populations, may further contribute to these discrepancies, making direct comparisons difficult.

The existing literature highlights a persistent gap between theoretical knowledge and practical readiness. While studies document varying levels of awareness, very few examine actual training participation rates or real-world first aid experience. In Saudi Arabia, Bashekah et al. found that only 55% of the general population possessed moderate first aid knowledge, with formal training rates remaining low [18]. This knowledge-practice gap is concerning, as evidence suggests that theoretical awareness without hands-on training does not translate into effective emergency response behavior [15,21]. Evidence suggests that questionnaire-assessed knowledge does not reliably reflect practical first aid performance [22] and that training-related gains in knowledge and confidence may diminish over time without reinforcement [23].

The southern regions of Saudi Arabia—including Jazan, Aseer, Al-Baha, and Najran—represent an understudied geographical area with unique demographic and healthcare characteristics. These regions combine urban centers with rural populations, varying educational levels, and distinct patterns of trauma epidemiology. Understanding public awareness and preparedness in this region is essential for developing targeted, culturally appropriate intervention programs. Furthermore, given the high burden of RTAs and the critical role of bystander first aid in determining outcomes, assessing the capacity of the public to recognize and respond appropriately to CSIs holds significant implications for trauma care systems and public health policy.

The current study seeks to address a notable gap in the literature by comprehensively examining: (1) public awareness about the recognition of cervical spine injuries, (2) knowledge of appropriate first aid responses, (3) rates of formal training participation, (4) real-world first aid experience, and (5) sociodemographic predictors of awareness among adult residents of southern Saudi Arabia. By examining both theoretical knowledge and practical preparedness, we aim to provide evidence-based recommendations for community education programs, policy interventions, and healthcare system improvements to enhance pre-hospital CSI management and ultimately reduce preventable morbidity and mortality from traumatic spinal cord injuries.

## 2. Materials and Methods

### 2.1. Study Design and Setting

Public awareness of cervical spine trauma, recognition of its warning signs and appropriate first aid responses among adult residents of the southern regions of Saudi Arabia were assessed through a cross-sectional study. Owing to its utility in measuring prevalence and awareness at a discrete time point, a cross-sectional design was utilized. Multi-variable data related to CSI awareness were subsequently gathered from the target population over the 2025–2026 period.

Data were drawn from four southern regions of Saudi Arabia, namely Jazan, Aseer, Al-Baha, and Najran. These regions were selected to provide comprehensive geographic coverage of the southern areas of the Kingdom, representing diverse demographic profiles including urban and rural populations with varying educational and socioeconomic characteristics.

### 2.2. Data Collection Instrument

The study questionnaire was developed through a rigorous systematic review of existing validated tools measuring public awareness of CSI and first aid practices [1,17,20]. The questionnaire was adapted from these established instruments to ensure content validity, relevance, and cultural appropriateness for the Saudi Arabian context. The adapted instrument underwent a multi-step validation process. Content validity was established through expert review by a panel in emergency medicine, physiotherapy, and public health, with minor wording modifications incorporated. A pilot test among 30 participants excluded from the main analysis required no major structural changes. Internal consistency was acceptable (Cronbach’s alpha = 0.73). As the instrument was administered in Arabic forward and back translation by two independent bilingual experts was performed, with discrepancies resolved by consensus. The final instrument comprised three main sections structured to comprehensively assess awareness, knowledge, and attitudes.

The first section collected sociodemographic data, including age, sex, nationality, education level, occupation, marital status, geographical residence (urban vs. rural), monthly income, employer type, body mass index (BMI), smoking status, and presence of chronic medical conditions. Comprising 20 items, the second section assessed participants’ knowledge of CSI across key domains, including risk factors, consequences, etiology, clinical presentation, and initial management principles, including immobilization and spinal motion restriction. Each item was scored dichotomously (correct = 1, incorrect = 0), yielding an awareness score ranging from 0 to 20 points. Scores ≥12 points (≥60%) were categorized as good awareness, while scores <12 points (<60%) were classified as poor awareness. The last section gathered information on participants’ attitudes toward first aid, willingness to assist trauma victims, training history, and previous experience with CSI.

### 2.3. Data Collection Process and Sampling

Between 2025 and 2026, data collection was carried out digitally via WhatsApp, Telegram, and X (formerly Twitter), selected for their high penetration rates in Saudi Arabia and their capacity to reach diverse demographic groups across geographic boundaries. The research team distributed standardized instructions with the questionnaire link and remained accessible to participants throughout the collection period.

**Figure 1:**
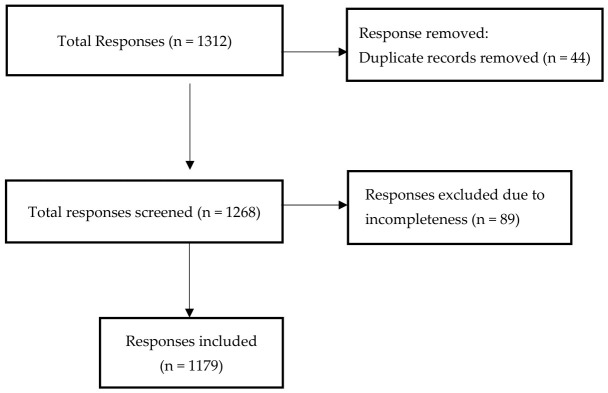
Participant flow diagram showing response screening and final study inclusion.

Participant recruitment targeted adults (≥18 years of age) through a convenience sampling strategy; it targeted those residing in the southern regions of Saudi Arabia who provided informed consent and had access to a smartphone or internet-enabled device capable of completing an online survey. Eligibility was restricted to adults residing in the four southern provinces (Jazan, Aseer, Al-Baha, or Najran), and all data collectors were based in these provinces and distributed the questionnaire through local networks within this catchment area. The questionnaire did not include a separate item identifying which of the four provinces each respondent resided in; consequently, province-level distribution within the southern region could not be quantified. Individuals who did not meet the inclusion criteria or declined to participate were excluded. Participants received clear instructions regarding questionnaire completion and could contact the research team for clarification at any time, ensuring accurate responses and data reliability. Validated instruments and periodic checks for redundant or incomplete entries formed the basis of quality control. Duplicate submissions were identified based on identical IP addresses combined with matching sociodemographic responses submitted within the same session timeframe. Due to the open-distribution nature of social media recruitment, an exact response rate could not be calculated; however, all completed responses were included in the final analysis after excluding incomplete submissions (Figure 1).

### 2.4. Sample Size

Sample size estimation was based on standard cross-sectional survey methodology using the formula:

n = (DEFF × Z^2^ × P × (1 − P))/d^2^

where n = sample size; DEFF = design effect (1); Z = 1.96 (95% confidence); P = 50% (conservative CSI awareness estimate); and d = 5% margin of error. The minimum required sample was 385; however, the target was raised to 1000 to enhance precision and enable subgroup analyses. Finally, 1179 participants completed the survey and were incorporated into the analysis.

### 2.5. Statistical Analysis

R software (version 4.3.0; R Foundation for Statistical Computing, Vienna, Austria) was employed for all statistical computations. The primary outcome variable was the overall awareness score, analyzed as both a continuous variable (range 0–20 points). 1. Sociodemographic characteristics including age, sex, nationality, educational attainment, occupation, marital status, place of residence, and monthly income, 2. Training-related variables, including participation in medical training courses, previous experience with CSI, were incorporated as predictor variables. Employer type was excluded from the multivariable model as it is nested within occupation and applies only to employed participants, introducing conceptual overlap and redundancy. Chronic medical conditions were examined in preliminary analyses but excluded from the final model due to low prevalence and absence of meaningful unadjusted associations.

All variables were subjected to descriptive statistical analysis. Frequencies and percentages were used to summarize categorical variables, whereas continuous variables depicted as means ± SDs. To pinpoint independent awareness score predictors, multivariable linear regression was performed with simultaneous entry of all variables and adjustment for confounders, generating β coefficients, 95% CIs, and *p*-values. Model fit diagnostics included assessment of residual distribution, calculation of variance inflation factors (VIF) to detect multicollinearity (VIF < 3 considered acceptable), and determination of R^2^ to quantify explained variance. Statistical significance was defined as *p* < 0.05 (two-tailed).

### 2.6. Ethical Considerations

The study received ethical clearance from the Local Research Ethics Committee at Jazan University (HAPO-10-Z-001, Ref. No. REC-46/09/1420, 5 March 2024), with all procedures conforming to institutional standards and the Declaration of Helsinki (1964) and its subsequent amendments. Before participation, electronic informed consent was secured, and participants were briefed on the study’s objectives, voluntary involvement, data privacy, and withdrawal rights. No personally identifiable information was collected, and all data was safely stored with restricted access.

### 2.7. Use of Generative Artificial Intelligence

ChatGPT-5 (OpenAI), which was only used for language editing in this study. All other elements, including study design, literature synthesis, data extraction, analysis, and interpretation, were carried out exclusively by the authors.

## 3. Results

### 3.1. Sociodemographic Characteristics of Participants

A total of 1179 participants were included in the study. The mean age was 30 ± 11 years, the mean weight was 69 ± 19 kg, the mean height was 166 ± 9.9 cm, and the mean body mass index (BMI) was 25 ± 6 kg/m^2^. Females comprised 44% of the sample. Most participants were Saudi nationals (96%). Regarding marital status, 62% were single, 37% were married, and 1% were divorced. Most participants resided in urban areas (62%), while 38% lived in rural areas. In terms of education, 70% held a bachelor’s degree, 25% had a high school education or less, and 5% were postgraduates. Regarding employment status, 36% were employed, and 15% were unemployed. Among employed participants, 41% were students, followed by those working in the educational sector (15%), healthcare sector (14%), military sector (7%), administrative sector (4%), industrial sector (4%), and other sectors (15%). Monthly income was less than 5000 SR for 57%, 5000–9999 SR for 17%, 10,000–14,999 SR for 11%, and more than 15,000 SR for 16%. Regarding smoking status, 78% had never smoked, 13% were current smokers, and 9% were former smokers (Table 1).

### 3.2. Health-Related Characteristics

Health-related characteristics are presented in Table 2. Hypercholesterolemia was the most frequently reported condition (9%), followed by asthma (7%), diabetes mellitus (6%), and hypertension (6%). Hypothyroidism was reported by 3% of participants. Sickle cell anemia, hyperthyroidism, rheumatoid arthritis, and hernia were each reported by 2%, while thalassemia was reported by 1% of participants.

### 3.3. Spinal Trauma and Life Support Training

**Figure 2:**
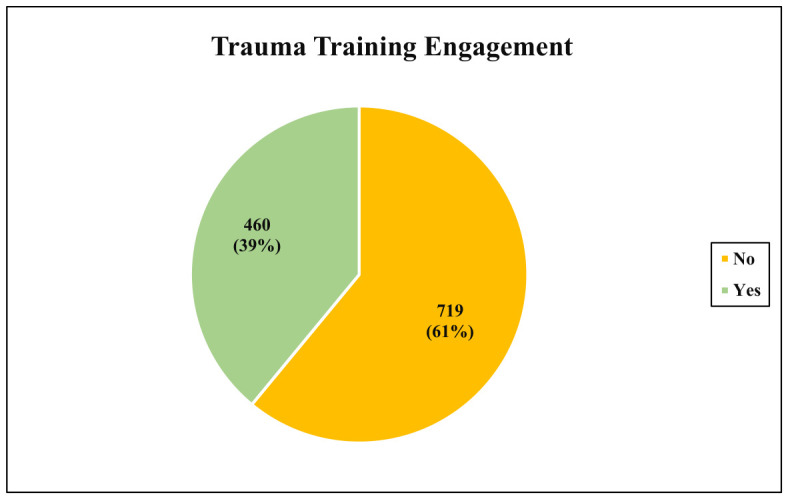
Distribution of participants based on engagement in trauma training programs.

**Figure 3:**
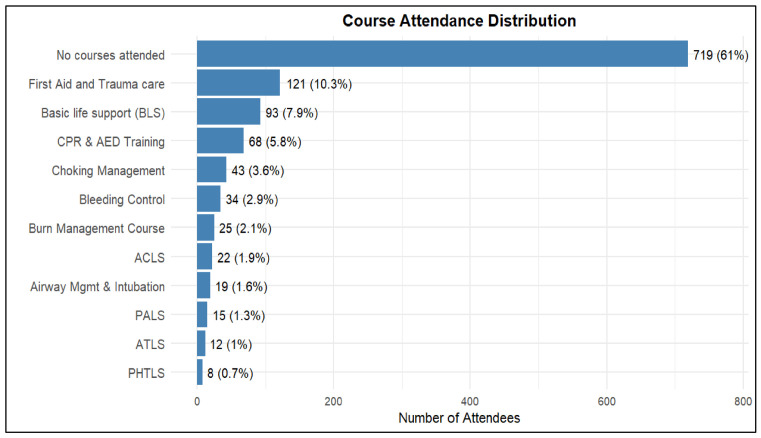
Distribution of participants by type of trauma and life support training program.

As shown in Table 3, 39% of participants attended medical training related to trauma injuries or emergencies, while 61% did not (Figure 2). Only 3% reported having experienced or witnessed a CSI, and the same proportion (3%) reported having provided first aid to an individual with a CSI. Figure 1 shows the distribution of participants based on engagement in trauma training programs. Among those who received training (39%), the most common courses were First Aid and Trauma Care (10.3%), Basic Life Support (BLS) (7.9%), and CPR & AED training (5.8%). Participation in advanced courses such as ACLS, ATLS, PALS, and PHTLS was low (≤1.9%), as illustrated in Figure 3.

### 3.4. Awareness and Practices Toward Spinal Injury

The mean awareness score was 16 ± 4.8, with 87% of participants classified as having good awareness (≥60%) and 13% as having poor awareness. Most participants (70%) expressed willingness to assist individuals involved in RTAs or falls. Furthermore, 72% believed that appropriate first aid responses could benefit victims, and 75% recognized that CSI could have long-term consequences, including death. Regarding emergency responses, 89% reported they would implement spinal motion precautions for an unconscious patient, 89% would avoid unnecessary movement while contacting emergency services, 93% would contact emergency services before initiating first aid, and 95% would maintain proper head–neck–spine alignment. In a scenario involving a responsive patient with suspected CSI, 83% selected the correct response (providing reassurance and instructing the patient to remain still) (Table 4).

### 3.5. Predictors of Awareness Score

Multivariable linear regression analysis (Table 5) showed that participants who had received relevant training had significantly higher awareness scores compared with untrained participants (β = 1.39, 95% CI: 0.80–1.98, *p* < 0.001). Married participants also demonstrated higher awareness scores compared with single participants (β = 1.37, 95% CI: 0.45–2.29, *p* = 0.004). No other sociodemographic or health-related variables were independently associated with awareness.

## 4. Discussion

This multiregional study of 1179 adults from southern Saudi Arabia reveals a paradoxical finding: while 87% of participants demonstrated good theoretical awareness of CSI first aid principles, only 39% had attended formal training, and merely 3% had real-world first aid experience. This critical knowledge-practice gap represents both a public health challenge and an opportunity for targeted intervention. Our findings contribute new insights into regional variations in trauma preparedness and highlight the urgent need for structured, accessible community-based training programs.

### 4.1. High Awareness Despite Minimal Formal Training

Our finding that 87% of participants achieved good awareness scores (mean of 16.0 ± 4.8 out of a possible 20 points, corresponding to 80% of the total score) is encouraging and aligns with recent data from Dammam, where 90.4% demonstrated adequate knowledge [19]. However, this contrasts sharply with the 53.8% awareness rate reported in Makkah [20] and the nationwide finding by Al-Otaibi et al. that fewer than one-third of participants were aware of appropriate CSI first aid [1].

In comparison to the Makkah and Dammam studies [19,20], our study extends prior regional work by providing multiregional coverage across four southern regions, a larger sample, and multivariable regression analysis, enabling more robust identification of independent predictors of CSI awareness while accounting for confounders.

Several factors may explain these regional disparities. First, our study population was relatively educated (70% with bachelor’s degrees), predominantly urban (62%), and nearly half were students (49%)—demographics associated with greater health literacy and access to information through digital media and educational curricula.

The mechanisms underlying high awareness in the absence of formal training (only 39%) are unclear from our data; possible explanations may include informal information exposure, though this hypothesis requires further investigation. This has important implications for intervention design. While informal knowledge dissemination creates a foundation of awareness, it lacks hands-on practice, scenario-based decision-making, and skill reinforcement essential for effective emergency response. The discrepancy between our 87% theoretical awareness and 83% correct scenario-based responses, though still high, may suggest this limitation, though this cannot be confirmed from the current data. More concerning is the 3% rate of actual first aid provision, indicating that when faced with real emergencies, even theoretically knowledgeable individuals may hesitate or fail to act appropriately due to lack of confidence, fear of causing harm, or inability to translate knowledge into practice [15,21,24]. It is important, however, to acknowledge that direct methodological comparability with these prior studies is limited; for example, the Dammam [19], Makkah [20], and nationwide [1] investigations differed in sampling frames, recruitment methods, questionnaire instruments, awareness-score thresholds, and demographic composition. The observed variation in awareness rates should therefore be interpreted as indicative of regional and methodological heterogeneity rather than as strictly comparable estimates, and our findings are best viewed as complementary to rather than directly comparable with previous Saudi research.

### 4.2. The Training Paradox: Knowledge Without Practice

Perhaps the most clinically important finding of this study is the dramatic training gap. Only 39% of participants attended any trauma-related training, and among those, participation concentrated in basic courses: First Aid and Trauma Care (10.3%), Basic Life Support (7.9%), and CPR & AED training (5.8%). Advanced courses critical for healthcare professionals—ACLS, ATLS, PALS, and PHTLS—had participation rates below 2%. More strikingly, only 3% had provided first aid for a CSI, and an equal percentage had witnessed or experienced such an injury personally.

This pattern reveals a troubling disconnect: high awareness exists alongside minimal hands-on preparedness. Our regression analysis confirmed training as the strongest predictor of awareness (β = 1.39, *p* < 0.001), representing approximately a 7% improvement in scores. This effect size, while statistically robust, suggests that untrained individuals may possess substantial baseline knowledge—likely acquired through informal education—but lack the confidence. Structured training may provide additional practical competencies beyond theoretical knowledge, though this was not directly assessed in this study [21,25]. International evidence consistently demonstrates that scenario-based, hands-on training is superior to didactic knowledge transfer alone for developing effective emergency response behaviors [15]. The Saudi context appears no different.

Studies from Saudi Arabia and Jordan provide consistent evidence that prior first aid training significantly improves knowledge. In Hail City, trained individuals demonstrated higher knowledge across multiple domains (*p*=0.0001) [26], while in Jordan, trained participants had significantly better scores, with 76.78% identifying lack of training as the main barrier [27].

The low training rates may reflect systemic barriers: cost of courses, time constraints, lack of accessible training centers in rural areas, absence of workplace or school-based programs, or insufficient public health promotion. Notably, even among healthcare sector workers (14% of our sample), training rates were not universal, and the multivariable model found no significant advantage for healthcare workers after adjusting for training, suggesting that employment in healthcare alone does not guarantee trauma preparedness without formal education [19]. This finding challenges assumptions about professional preparedness and highlights the need for mandatory, recurring training even within health systems.

### 4.3. Sociodemographic Predictors: Beyond Training

Beyond training, only marital status emerged as an independent predictor of awareness, with married individuals scoring 1.37 points higher than single participants (*p* = 0.004). This may plausibly reflect greater health-related responsibility and information-seeking behavior associated with family roles such as caring for children, elderly parents, or spouses, as well as increased exposure to household safety concerns and greater engagement with primary healthcare services, all of which could heighten attention to first aid information. However, because these potential mechanisms were not directly measured, the observed association likely also reflects confounding by unmeasured variables such as age, parental status, or accumulated life experience. This finding should therefore be interpreted cautiously and warrants further investigation in future studies incorporating more detailed psychosocial and behavioral measures.

Notably, factors that we hypothesized would predict awareness, such as education level, healthcare sector employment, urban residence, income, age, and sex, showed no independent association after adjusting for training. This finding may suggest that training could reduce awareness disparities across sociodemographic groups, though causal conclusions cannot be drawn from this cross-sectional design. From a public health perspective, this is encouraging, as it indicates that well-designed, accessible training programs can reduce health literacy disparities and democratize emergency preparedness across diverse populations [14,18].

### 4.4. Clinical and Public Health Implications

The findings of this study have immediate practical applications. First, the high baseline awareness provides a strong foundation upon which to build hands-on training programs. Rather than starting from zero, educational interventions can leverage existing knowledge and focus on skill development, confidence-building, and scenario-based practice. Second, the demonstration that training is the primary modifiable predictor of awareness suggests that investment in accessible, affordable, community-based first aid courses could potentially yield measurable improvements in public preparedness. Third, the concentration of our sample in younger, educated demographics indicates that schools and universities represent high-yield settings for intervention—capturing individuals during formative years when behaviors and skills are most readily acquired [28,29].

Specific recommendations include: (1) integration of basic first aid and trauma response modules into secondary school and university curricula as mandatory components of health education; (2) workplace-based training programs, particularly in high-risk sectors (transportation, construction, sports, education) where traumatic injuries are more likely; (3) community outreach through primary healthcare centers, utilizing existing infrastructure for periodic public training sessions; (4) leveraging social media and digital platforms—which clearly reach this population—for awareness campaigns linked to practical training opportunities; and (5) policy advocacy for subsidized or free first aid certification programs, reducing financial barriers to participation [8,12,13].

For healthcare systems, these findings underscore the importance of pre-hospital care quality in determining trauma outcomes. While emergency medical services (EMS) response times in Saudi Arabia have improved, bystander actions in the critical minutes before EMS arrival can prevent secondary injury or stabilize patients for transport [16,17]. Strengthening the first link in the chain of survival—immediate bystander response—complements investments in ambulance services, trauma centers, and rehabilitation facilities. Given the Kingdom’s Vision 2030 emphasis on healthcare quality and preventive medicine, expanding public first aid competency aligns with national strategic priorities [9].

### 4.5. Strengths and Limitations

This study is strengthened by its large sample size (n = 1179), multiregional coverage, use of a validated questionnaire, inclusion of both knowledge- and scenario-based items, and assessment of training participation and real-world first aid practice, enabling evaluation of the knowledge–practice gap. As data collection was conducted exclusively online, individuals without smartphones or internet access were systematically excluded, potentially skewing the sample toward more digitally connected and educated participants and thereby overestimating population-level awareness. The use of multivariable regression allowed for the identification of independent predictors while controlling for confounders. However, the cross-sectional design limits causal inference, and convenience sampling via social media may have introduced selection bias toward younger, urban, and more educated participants, potentially overestimating awareness. Self-reported data are subject to social desirability bias, and the knowledge assessment did not include an objective evaluation of practical skills. Although all participants met the southern-residence inclusion criterion, the questionnaire did not capture which specific southern province (Jazan, Aseer, Al-Baha, or Najran) each respondent resided in. Province-stratified analyses were therefore not possible, and uneven representation across the four provinces cannot be excluded. Findings should be interpreted as reflecting the southern region collectively. Future studies should include an explicit province-of-residence item to enable stratified analysis of intra-regional variation. Training was not differentiated by quality or duration, and the relatively homogeneous, predominantly young Saudi sample limits generalizability. Finally, the study did not assess clinical or patient-level outcomes, which represent the ultimate public health impact of bystander first aid.

### 4.6. Future Research Directions

Future studies should employ longitudinal designs to evaluate the sustained impact of training interventions on knowledge retention and behavior change. Observational studies in emergency departments could assess the quality of pre-hospital cervical spine management and identify associations between bystander actions and patient outcomes. Mixed-methods research incorporating qualitative interviews would illuminate barriers to training participation and factors influencing bystander decision-making during actual emergencies. Intervention trials comparing different training modalities (online vs. in-person, brief vs. comprehensive, scenario-based vs. didactic) would inform optimal program design. Studies of older adults, rural populations, and non-Arabic speakers would address gaps in current evidence. Finally, health economic analyses evaluating the cost-effectiveness of population-based first aid training versus trauma care costs and disability-adjusted life years could strengthen the policy case for investment in public education programs [30].

## 5. Conclusions

Although 87% of adults in southern Saudi Arabia demonstrated good theoretical awareness of CSI first aid, this finding must be interpreted alongside the striking contrast of only 39% formal training participation and 3% real-world first aid experience, revealing a critical knowledge-practice gap that limits the public health value of awareness alone. Formal trauma training was the strongest independent predictor of awareness, identifying it as the primary modifiable target for intervention. While our data did not directly assess training preferences or barriers, these findings suggest that expanding access to hands-on first aid training represents the most evidence-informed strategy to bridge this gap. Future efforts should consider integrating structured trauma training into accessible community settings, with specific modalities and delivery platforms informed by dedicated needs-assessment research.

## Figures and Tables

**Table 1:** Sociodemographic characteristics of study participants (n = 1179).

Variable	*Mean ± SD*
Age	*30 ± 11 years*
Weight	*69 ± 19 kg*
Height	*166 ± 9.9 cm*
BMI	*25 ± 6*
*Characteristic*	
Sex	
*Female*	*514 (44%)*
*Male*	*665 (56%)*
Nationality	
*non-Saudi*	*48 (4%)*
*Saudi*	*1131 (96%)*
Marital Status	
*Single*	*728 (62%)*
*Married*	*435 (37%)*
*Divorced*	*16 (1%)*
Residence	
*Rural Area*	*450 (38%)*
*Urban Area*	*729 (62%)*
Education	
*High school or less*	*298 (25%)*
*Bachelor’s degree*	*825 (70%)*
*Postgraduate*	*56 (5%)*
Student	*483 (41%)*
Occupation	
*Employed*	*421 (36%)*
*Unemployed*	*177 (15%)*
Employer	
*Administration Sector*	*47 (4%)*
*Educational Sector*	*182 (15%)*
*Healthcare Sector*	*162 (14%)*
*Industrial Sector*	*45 (4%)*
*Military Sector*	*87 (7%)*
*Other*	*173 (15%)*
Monthly Income	
*Less than 5000 SR*	*674 (57%)*
*5000 to 9999 SR*	*196 (17%)*
*10,000 to 14,999 SR*	*124 (11%)*
*More than 15,000 SR*	*185 (16%)*
Smoking	
*Current*	*154 (13%)*
*EX-Smoker*	*102 (9%)*
*Never smoked*	*923 (78%)*

*Abbreviations: SD: Standard deviation n: Sample size SR: Saudi Riyal (1 SAR ≈ 0.27 USD) BMI: Body mass index*.

**Table 2:** Health-related characteristics of the sample (n = 1179).

*Characteristic*	*Frequency (%)*
Diabetes	
*No*	*1107 (94%)*
*Yes*	*72 (6%)*
Hypertension	
*No*	*1106 (94%)*
*Yes*	*73 (6%)*
Hypercholesterolemia	
*No*	*1076 (91%)*
*Yes*	*103 (9%)*
Asthma	
*No*	*1091 (93%)*
*Yes*	*88 (7%)*
Sickle cell anemia	
*No*	*1156 (98%)*
*Yes*	*23 (2%)*
Thalassemia	
*No*	*1169 (99%)*
*Yes*	*10 (1%)*
Hyperthyroidism	
*No*	*1160 (98%)*
*Yes*	*19 (2%)*
Hypothyroidism	
*No*	*1145 (97%)*
*Yes*	*34 (3%)*
Rheumatoid arthritis	
*No*	*1152 (98%)*
*Yes*	*27 (2%)*
Hernia	
*No*	*1153 (98%)*
*Yes*	*26 (2%)*

**Table 3:** Spinal trauma and Life support Training variables (n = 1179).

*Characteristic*	*Frequency (%)*
Have you ever attended medical training courses on handling injuries and emergencies?	
*No*	*719 (61%)*
*Yes*	*460 (39%)*
Have you ever had or witnessed any CSI?	
*No*	*1138 (97%)*
*Yes*	*41 (3%)*
Have you ever provided first aid care to an individual with a CSI?	
*No*	*1142 (97%)*
*Yes*	*37 (3%)*

**Table 4:** Awareness and practices toward spinal injury (n = 1179).

Variable	Mean ± SD
Awareness score	16 *±* 4.8
Awareness category	***Frequency (%)***
good	*1026 (87%)*
poor	*153 (13%)*
*Characteristic*	***Frequency (%)***
I am willing to assist individuals who are involved in a car accident or a fall.	
*No*	*352 (30%)*
*Yes*	*827 (70%)*
An appropriate response can be beneficial for the victim.	
*I don’t know*	*295 (25%)*
*No*	*39 (3%)*
*Yes*	*845 (72%)*
A CSI can affect the patient’s quality of life until their death.	
*No*	*46 (4%)*
*I don’t know*	*253 (21%)*
*Yes*	*880 (75%)*
I would implement spinal movement precautions for an unconscious patient with trauma.	
*No*	*126 (11%)*
*Yes*	*1053 (89%)*
I will avoid moving the patient and promptly call for emergency services.	
*I don’t know*	*87 (7%)*
*No*	*39 (3%)*
*Yes*	*1053 (89%)*
I will contact emergency services before initiating first aid measures.	
*No*	*85 (7%)*
*Yes*	*1094 (93%)*
I will consistently exercise utmost caution to keep the head, neck, and spine properly aligned.	
*No*	*54 (5%)*
*Yes*	*1125 (95%)*
If the patient is responsive and I suspect a CSI, I will take the following steps.	
*Check the patient’s neck mobility*	*38 (3%)*
*I don’t know*	*145 (12%)*
*Provide reassurance and tell the patient to remain still*	*982 (83%)*
*Try to change the patient’s position*	*14 (1%)*

**Table 5:** Predictors of Awareness Score Among Participants.

	Awareness Score		
*Predictor*	*Estimate*	*CI*	*p*
*(Intercept)*	13.92	11.05–16.79	**<0.001**
*Training [Yes]*	1.39	0.80–1.98	**<0.001**
*Sex [Male]*	−0.29	−0.94–0.36	0.381
*Nationality [Saudi]*	0.19	−1.20–1.58	0.788
*Age*	0.02	−0.03–0.06	0.420
*BMI*	0.03	−0.02–0.08	0.226
*Marital Status [Divorced]*	0.99	−1.40–3.39	0.417
*Marital Status [Married]*	1.37	0.45–2.29	**0.004**
*Residence [Urban Area]*	−0.33	−0.90–0.23	0.246
*Education [College]*	−0.03	−0.70–0.63	0.922
*Education [Postgraduate]*	−0.31	−1.76–1.13	0.669
*Occupation [Employed]*	−0.51	−1.70–0.67	0.397
*Occupation [Student]*	0.19	−0.96–1.34	0.749
*Monthly Income [10,000 to* *14,999 SR]*	−0.22	−1.35–0.92	0.709
*Monthly Income [5000 to* *9999 SR]*	0.20	−0.74–1.15	0.673
*Monthly Income [More than* *15,000 SR]*	0.26	−0.76–1.27	0.619
*Smoking [Current]*	0.39	−0.47–1.25	0.373
*Smoking [EX- Smoker]*	0.08	−0.94–1.09	0.880
*Observations*	1179		

## Data Availability

The datasets generated and/or analyzed during the current study are available from the corresponding author on reasonable request. The data are not publicly available due to ethical and privacy restrictions involving sensitive health-related information.

## Abbreviations

The following abbreviations are used in this manuscript:

CSIs	Cervical Spine Injuries
RTAs	Road Traffic Accidents
EMS	Emergency Medical Services
CPR	Cardiopulmonary Resuscitation
ACLS	Advanced Cardiovascular Life Support
ATLS	Advanced Trauma Life Support
PALS	Pediatric Advanced Life Support
PHTLS	Prehospital Trauma Life Support
SD	Standard Deviation

## References

[1] Al-Otaibi M.L., Almutairi K.H., Al-Otaibi K.M., Alghaeb A.N., Al-Hadi S.H. (2021). Levels of public awareness regarding cervical spine injury and the suitable first aid response among adults in Saudi Arabia. Saudi Med. J..

[2] Cramer G.D., Darby S.A. (2013). Clinical Anatomy of the Spine, Spinal Cord, and ANS.

[3] Sarkodie-Gyan T., Yu H. (2023). The human locomotor system: Physiological and technological foundations. The Human Locomotor System: Physiological and Technological Foundations.

[4] Mahadevan V. (2018). Anatomy of the vertebral column. Surgery.

[5] McElhaney J.H., Myers B.S. (1993). Biomechanical aspects of cervical trauma. Accidental Injury: Biomechanics and Prevention.

[6] Lu Y., Shang Z., Zhang W., Pang M., Hu X., Dai Y., Rong L. (2024). Global incidence and characteristics of spinal cord injury since 2000–2021: A systematic review and meta-analysis. BMC Med..

[7] Kim M., Jeong W., Jang S., Park J.H., Bae Y., Lee S.W. (2025). Spinal Cord Injury Epidemiology and Causes: A Worldwide Analysis with 2050 Projections. Healthcare.

[8] World Health Organization (2013). Global Status Report on Road Safety 2013: Supporting a Decade of Action.

[9] GBD 2017 Saudi Arabia Collaborators (2020). *The burden of disease in Saudi Arabia* 1990–2017: Results from the Global Burden of Disease Study 2017. Lancet Planet. Health.

[10] Shanks N.J., Ansari M., al-Kalai D. (1994). Road traffic accidents in Saudi Arabia. Public. Health.

[11] Ghaffar U.B., Ahmed S. (2015). A review of road traffic accident in Saudi Arabia: The neglected epidemic. Indian. J. Forensic Community Med..

[12] Aldwsari O.M., Aldosari K.H., Alzahrani M.K., Alzahrani Z.A., Alanazi A.H., Alkhathlan K.M., Al-Ghamdi S. (2018). Associated head injuries and survival rate of patients with maxillofacial fractures in road traffic accident: A prospective study in Saudi Arabia. J. Fam. Med. Prim. Care.

[13] Mokdad A.H., Jaber S., Aziz M.I.A., AlBuhairan F., AlGhaithi A., AlHamad N.M., Murray C. (2014). JThe state of health in the Arab world, 1990–2010: An analysis of the burden of diseases, injuries, and risk factors. Lancet.

[14] Khan A., Shaikh S., Shuaib F., Sattar A., Samani S.A., Shabbir Q., Rasheed A.Z. (2010). Knowledge, attitude and practices of undergraduate students regarding first aid measures. J. Pak. Med. Assoc..

[15] Kureckova V., Gabrhel V., Zamecnik P., Rezac P., Zaoral A., Hobl J. (2017). First aid as an important traffic safety factor—Evaluation of the experience-based training. Eur. Transp. Res. Rev..

[16] Vaillancourt C., Charette M., Kasaboski A., Maloney J., Wells G.A., Stiell I.G. (2011). Evaluation of the safety of C-spine clearance by paramedics: Design and methodology. BMC Emerg. Med..

[17] Yisheng W., Fuying Z., Limin W., Junwei L., Guofu P., Weidong W. (2007). First aid and treatment for cervical spinal cord injury with fracture and dislocation. Indian. J. Orthop..

[18] Bashekah K.A., Alqahtani R., Aljifri A.M., Ashram S.Y., Alghamdi E., Khallaf A.M., Banaja A. (2023). The knowledge, attitudes, and associated factors regarding first aid among the general public in Saudi Arabia. Cureus.

[19] Al-Othman A.M., Alatawi F.H., Alshaikhi O.M. (2018). Assessment of knowledge, attitude and practice towards cervical-spinal injury among adults in Dammam City, 2017. Egypt. J. Hosp. Med..

[20] Alghamdi F.A., Alghamdi G.A., Almatrafi W.S., Alghamdi R.F., Kelantan S.R., Abdulsamad F.F. (2024). Public awareness levels regarding cervical spine injury and the suitable first aid response among adults in Makkah, Saudi Arabia. Int. J. Gen. Med..

[21] Williams G.N., Arciero R.A., Taylor D.C. (2018). Knowledge and preparedness of professionals managing spinal injuries. J. Athl. Train..

[22] Minna S., Leena H., Tommi K. (2022). How to evaluate first aid skills after training: A systematic review. Scand. J. Trauma. Resusc. Emerg. Med..

[23] Bakke H.K., Steinvik T., Eidissen S.I., Gilbert M., Wisborg T. (2015). Bystander first aid in trauma-prevalence and quality: A prospective observational study. Acta Anaesthesiol. Scand..

[24] Bangash M.H., Saeedi R., Al-Ghamdi W.A. (2019). The Saudi public’s knowledge level of spinal injury: A novel risk prediction scoring system. Int. J. Med. Res. Health Sci.

[25] Williams R.M., Welch Bacon C.E., Kucera K.L., Snyder Valier A.R. (2019). Athletic trainers’ knowledge of appropriate care of spine injured athletes. Athl. Train. Sports Health Care.

[26] Alshammari K.O. (2021). Assessment of knowledge, attitude, and practice about first aid among male school teachers in Hail city. J. Fam. Med. Prim. Care.

[27] Al-Qerem W., Jarab A., Al Bawab A.Q., Hammad A., Eberhardt J., Alasmari F., Kalloush H., Al-Sa’di L., Obidat R. (2024). An Online-Based Survey to Assess Knowledge, Attitudes, and Barriers to Perform First Aid after Road Accidents Conducted among Adult Jordanians. Healthcare.

[28] Wilberger J.E. (2000). Athletic cervical spinal cord and spine injuries. Neurologic Athletic Head and Spine Injuries.

[29] Tawakul A., Samkari J., Babateen O., Alzubaidi B., Alhuzali M., Alshinkity M., Alnashri A. (2024). Awareness of spinal cord injury among medical students at Umm Al-Qura University in Makkah City, Saudi Arabia: A cross-sectional study. J. Health Sci..

[30] Zaveri G., Das G. (2017). Management of sub-axial cervical spine injuries. Indian. J. Orthop..

